# Structural and *In Vitro* Functional Analyses of Novel Plant-Produced Anti-Human PD1 Antibody

**DOI:** 10.1038/s41598-019-51656-1

**Published:** 2019-10-23

**Authors:** Kaewta Rattanapisit, Tanapati Phakham, Supranee Buranapraditkun, Konlavat Siriwattananon, Chatikorn Boonkrai, Trairak Pisitkun, Nattiya Hirankarn, Richard Strasser, Yoshito Abe, Waranyoo Phoolcharoen

**Affiliations:** 1 Plant-Produced Pharmaceuticals Research Unit, Chulalongkorn University, 254 Phyathai Road, Patumwan, Bangkok 10330 Thailand; 2 Pharmacognosy and Pharmaceutical Botany Department, Faculty of Pharmaceutical Sciences, Chulalongkorn University, 254 Phyathai Road, Patumwan, Bangkok 10330 Thailand; 3 Interdisciplinary Program of Biomedical Sciences, Graduate School, Chulalongkorn University, Bangkok, Thailand; 4 Center of Excellence in Systems Biology, Research affairs, Faculty of Medicine, Chulalongkorn University, Bangkok, Thailand; 5 Division of Allergy and Clinical Immunology, Department of Medicine, Faculty of Medicine, Chulalongkorn University, Bangkok, Thailand; 6 Center of Excellence in Vaccine Research and Development (Chula Vaccine Research Center-Chula VRC), Faculty of Medicine, Chulalongkorn University, Bangkok, 10330 Thailand; 7 Department of Applied Genetics and Cell Biology, University of Natural Resources and Life Sciences, Vienna, Muthgasse 18, 1190 Vienna, Austria; 8 Laboratory of Protein Structure, Function and Design, Graduate School of Pharmaceutical Sciences, Kyushu University, 3-1-1 Maidashi, Higashi-Ku, Fukuoka 812-8582 Japan

**Keywords:** Synthetic biology, Molecular engineering in plants

## Abstract

Immunotherapy has emerged as a promising and effective treatment for cancer. The frequently used immunotherapy agents are immune checkpoint inhibitors, such as antibodies specific to PD1, PD-L1, or CTLA-4. However, these drugs are highly expensive, and most people in the world cannot access the treatment. The development of recombinant protein production platforms that are cost-effective, scalable, and safe is needed. Plant platforms are attractive because of their low production cost, speed, scalability, lack of human and animal pathogens, and post-translational modifications that enable them to produce effective monoclonal antibodies. In this study, an anti-PD1 IgG4 monoclonal antibody (mAb) was transiently produced in *Nicotiana benthamiana* leaves. The plant-produced anti-PD1 mAb was compared to the commercial nivolumab produced in CHO cells. Our results showed that both antibodies have similar protein structures, and the N-glycans on the plant-produced antibody lacks plant-specific structures. The PD1 binding affinity of the plant-produced and commercial nivolumab, determined by two different techniques, that is, enzyme-linked immunosorbent assay (ELISA) and surface plasmon resonance (SPR), are also comparable. Plant-produced nivolumab binds to human PD1 protein with high affinity and specificity, blocks the PD-1/PD-L1 interaction, and enhances T cell function, comparable to commercial nivolumab. These results confirmed that plant-produced anti-PD1 antibody has the potential to be effective agent for cancer immunotherapy.

## Introduction

Immunotherapy is currently a promising cancer treatment that activates the immune system and alleviates the suppression of the immune system by the tumour. The blockade of immune checkpoints is one of the most efficient approaches for activating anti-tumour immunity. There are 7 immune checkpoint inhibitors approved by USFDA^1^ including nivolumab, pembrolizumab, atezolizumab, durvalumab, avelumab, cemiplimab, and ipilimumab. However, these drugs are highly expensive^2,3^. Most people cannot afford to access this treatment. Therefore, it is necessary to develop novel platforms that are cost-effective, scalable, and safe.

Comparing to current antibody production platforms, plants offer several advantages for producing antibodies over others. Plants have significantly lower infrastructure and raw material cost for upstream production compared to mammalian cells^4,5^. Plants-based system can do greater scalability because there is no restriction on the fermenter size. The scalability can increase easily by growing more plants^6^. For the speed of production, transient plant-based technology can produce large amount of protein rapidly^7^. Moreover, antibodies produced in plants are not prone to contamination with human or animal pathogens. Plant cells efficiently assemble complexed proteins, such as antibodies, and perform necessary posttranslational modifications^8^. Plant-produced recombinant proteins were shown to meet the same good manufacturing practice criteria as proteins produced in mammalian cells, such as appearance, stability, half-life, immunogenicity etc^9^. These advantages for plant platforms suggest that they represent an attractive alternative for producing biopharmaceuticals for developing countries^10,11^.

In this study, we further developed an anti-PD1 monoclonal antibody (mAb), nivolumab, as a possible cancer immunotherapeutic by applying plant technology that enables cost-effective and scalable antibody production. We produced nivolumab at 140 μg/g fresh *Nicotiana benthamiana* leaves within 6 days of infiltration. Plant-produced nivolumab retained similar structure and *in vitro* efficacy, compared to those of commercial mammalian cell-produced nivolumab. This study is the first to demonstrate the use of plant-derived mAb for cancer immunotherapy.

## Results

### Expression and purification of anti-PD1 monoclonal antibodies in ΔXF *N. benthamiana*

**Figure 1:**
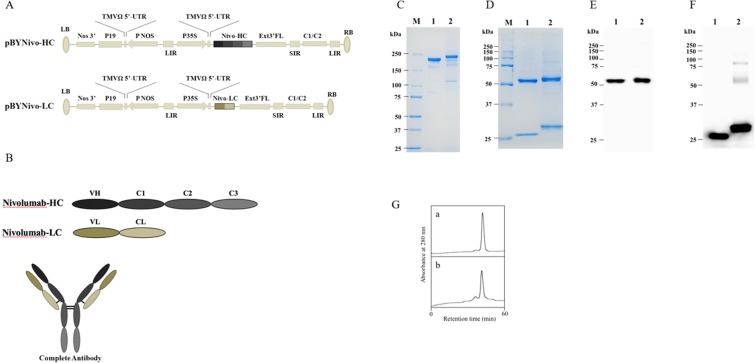
Plant-produced nivolumab antibody. (**A**) Schematic representation of the T-DNA regions of the vectors used in this study. (**B**) Schematic representation of nivolumab heavy chain (HC), light chain (LC) and completed antibody. Purified plant-produced nivolumab antibody in SDS-PAGE under non-reducing condition (**C**), reducing condition (**D**). Western blot analysis of plant-produced nivolumab antibody under reducing condition and probed with anti-human IgG gamma chain conjugated with HRP (**E**) or anti-human IgG kappa chain conjugated with HRP (**F**) M: protein ladder, 1: commercial nivolumab antibody 2: plant-produced nivolumab antibody. (**G**) Elution profiles of size-exclusion chromatography of (a) commercial nivolumab antibody and (b) plant-produced nivolumab antibody.

**Figure 2:**
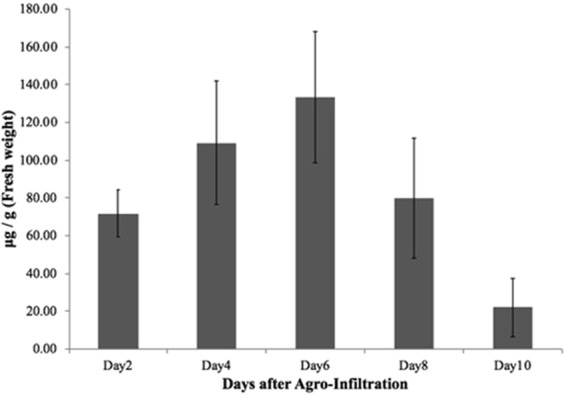
Transient expression of nivolumab antibody in *Nicotiana benthamiana* plants. Leaves were collected on days 2, 4, 6, 8 and 10 after agroinfiltration and quantified with ELISA. The expression level of plant-produced nivolumab was shown in micrograms of Nivolumab antibody per gram fresh weight. Data are means ± SD of samples from three independent infiltration experiments.

To increase the yield of monoclonal antibody (mAb) expression in plants, the coding sequences of the heavy chain (HC) and light chain (LC) of nivolumab^12^ were optimized *in silico* with *N. benthamiana*-optimized codon, cloned into geminiviral vectors and transformed into *Agrobacterium tumefaciens*. *A. tumefaciens* harbouring pBYNivo-HC and pBYNivo-LC (Fig. 1A) were co-delivered into *N. benthamiana* leaves through vacuum infiltration. Co-expression of the HC and LC resulted in the assembly of the complete antibody consisting of 2HC and 2LC (Fig. 1B). The expressed antibody was purified using protein A affinity chromatography and subjected to SDS-PAGE. The nonreducing SDS-PAGE confirmed that the complete mAb assembled into its tetrameric form (Fig. 1C). The reducing gel electrophoresis confirmed that the HC and LC of anti-PD1 mAb were expressed in leaves with the expected molecular weights (Fig. 1D). Notably, differences in mobility are caused by the presence of the SEKDEL peptide on the HC and LC. Western blot analysis after reducing gel electrophoresis confirmed the expression of HC and LC with anti-human gamma and anti-human kappa antibodies (Fig. 1E,F, respectively). Moreover, the purified antibody was analyzed by size exclusion chromatography. The result confirmed that the purified plant-produced monoclonal antibody remains monomer as commercial nivolumab (Fig. 1G). The assembly of anti-PD1 mAb was confirmed by an ELISA that detects the assembled form of the antibody. ELISA results also showed that the anti-PD1 mAb was expressed at the highest levels on 6 dpi, producing approximately 140 µg/g leaf fresh weight (Fig. 2).

### Plant-produced anti-PD1 exhibited mammalian-like N-linked glycosylation patterns

**Figure 3:**
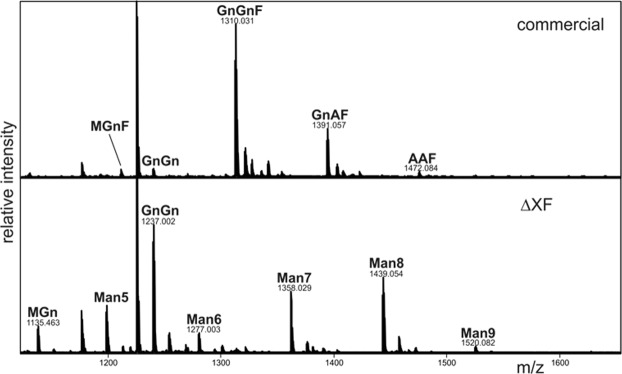
LC-ESI-MS of trypsin digested heavy chain from commercial nivolumab (commercial) and nivolumab produced in ΔXF *N. benthamiana* plants (ΔXF). The N-glycosylation profile of the glycopeptide EEQFNSTYR is shown, and the major glycosylated peaks [M + 2H]^+^ are depicted. MGnF: GlcNAcMan_3_FucGlcNAc_2_; GnGn: GlcNAc_2_Man_3_GlcNAc_2_; GnGnF: GlcNAc_2_Man_3_FucGlcNAc_2_; GnAF: GalGlcNAc_2_Man_3_FucGlcNAc_2_; AAF: Gal_2_GlcNAc_2_Man_3_FucGlcNAc_2_; MGn: GlcNAcMan_3_GlcNAc_2_; Man5-Man9: Man_5–9_GlcNAc_2_. For a detailed explanation of glycan structure abbreviations, see www.proglycan.com.

The N-glycan composition of the plant-produced nivolumab was compared to commercial CHO cell-produced antibody by liquid chromatography-electrospray ionization-mass spectrometry (LC-ESI-MS) analysis of tryptic glycopeptides (Fig. 3). As expected, the results confirmed that the N-glycan composition of nivolumab produced in ΔXF plants contained mainly GnGn (GlcNAc_2_Man_3_GlcNAc_2_) structures that more closely resemble mammalian-type N-glycans. As expected, GnGnF (GlcNAc_2_Man_3_FucGlcNAc_2_) carrying the mammalian-specific core fucosylation was the major N-glycosylated peptide in the commercial CHO cell-produced antibody (Fig. 3).

### Secondary and tertiary structural characterization

**Figure 4:**
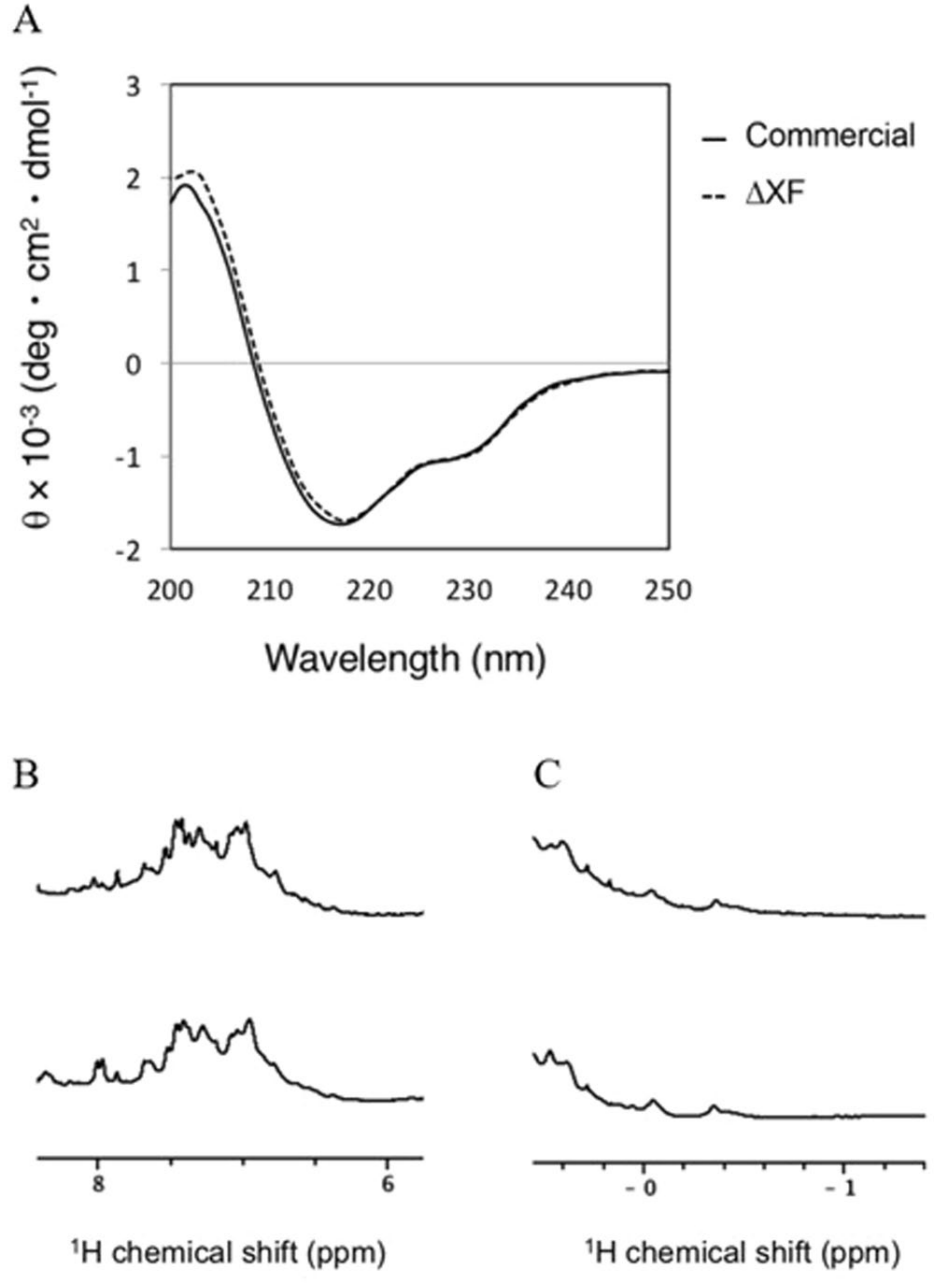
Structural analysis of plant-produced anti-PD1 and commercial mammalian cell-produced antibodies. (**A**) Plant-produced anti-PD1 antibody (solid line) and commercial mammalian cell-produced antibody (dashed line) in PBS (pH 7.4) were scanned by a circular dichroism spectrometer from 200–250 nm at room temperature. Both proteins exhibited a maximum wavelength at 218 nm. (**B,C**) NMR spectra in (**B**) aromatic region and (**C)** methyl region of commercial mammalian cell produced antibody (upper) and plant-produced anti-PD1 (lower) in PBS buffer (pH 7.4) containing 10% v/v D_2_O at 25 °C are shown, respectively.

The secondary and tertiary structures of plant-produced anti-PD1 were compared to commercial mammalian cell-produced antibody using circular dichroism (CD) and nuclear magnetic resonance (NMR) spectroscopy, respectively. The CD results (Fig. 4A) confirmed that the secondary structure of plant-produced anti-PD1 is similar to that of the commercial antibody. Both spectra indicate negative absorbance at 218 nm, which is a β-sheet-rich structure. For NMR spectroscopy, dispersed aromatic protons (Fig. 4B) and up-fielded methyl protons (Fig. 4C) were observed in both spectra, indicating that the tertiary structures are retained. Furthermore, both NMR spectra also confirmed that the tertiary structure of the plant-produced anti-PD1 antibody is similar to that of the commercial antibody.

### Functional assays of plant-produced anti-PD1 mAb

**Figure 5:**
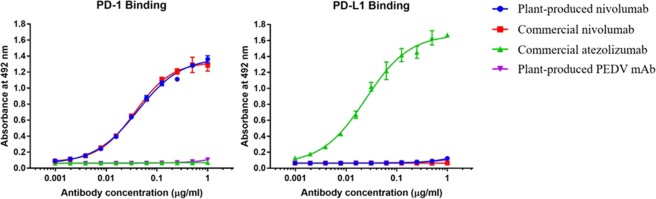
Specific binding to human PD-1 is confirmed by indirect ELISA. Serial dilutions of monoclonal antibodies (Plant-produced nivolumab, Commercial nivolumab, Commercial atezolizumab, Plant-produced PEDV mAb) were incubated on plates coated with human PD-1 (**A**) or human PD-L1 (**B**) proteins and detected with HRP-conjugated anti-human IgG antibody. Commercial atezolizumab (anti-PD-L1)^30^ and plant-produced PEDV mAb^14^ were used as negative control in PD1 binding assay (**A**). Commercial atezolizumab was used as positive control in PDL1 binding assay (**B**). The absorbance at 492 nm (mean ± SD) from triplicates are presented.

The *in vitro* binding to human PD1 protein of the plant-produced anti-PD1 mAb was determined by ELISA, as shown in Fig. 5A. Both plant cell-produced and commercial mammalian cell-produced anti-PD1 mAbs showed similar binding to human PD1 protein. Atezolizumab (anti-PDL1 mAb)^13^ and 2C10 (anti-Porcine Epidemic Diarrhea virus (PEDV) mAb)^14^ were used as the negative control. Both anti-PD1 mAbs could not bind to human PDL1 protein (Fig. 5B).

**Figure 6:**
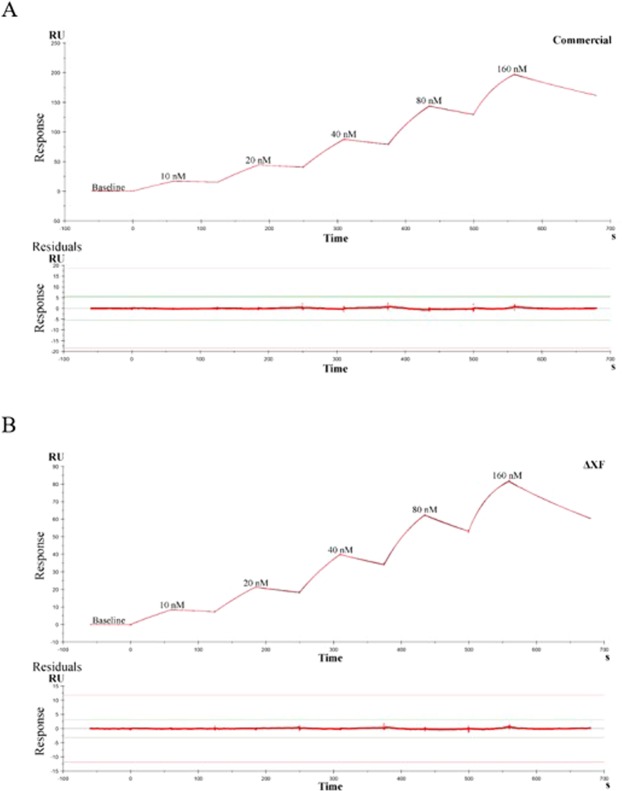
Binding affinity measurement of antibodies to human PD-1 protein by SPR (Biacore T200). Sensorgrams of (**A**) commercial nivolumab (commercial) and (**B**) nivolumab produced in ΔXF *N. benthamiana* plants (ΔXF). Human PD-1 protein was injected into the flow cell at various concentrations (10 nM-160 nM) to evaluate the binding/kinetics constants. The equilibrium dissociation constant (K_D_) was calculated using a 1: 1 Langmuir-binding model.

To assess the binding of the antibody more quantitatively, a surface plasmon resonance (SPR) assay was performed with purified plant-produced or commercial mammalian cell-produced anti-PD1 mAb immobilized on a Protein G sensor chip. The human PD1 protein was generated and flowed across the solid-phase anti-PD1 mAb at various concentrations. Plant-produced anti-PD1 mAb showed similar binding affinity and kinetics for human PD1 protein compared to commercial mammalian cell-produced mAb (Fig. 6). The association rate constant k_a_, dissociation rate constant k_d_, and equilibrium dissociation constant K_D_ of the plant-produced and commercial nivolumab are detailed in Table 1. The K_D_ between human PD1 and the plant-produced and commercial antibodies are 14 nM and 12 nM, respectively (Table 1). This data confirmed that both mAbs can bind to human PD1 protein with high affinity similarly.

**Table 1:** Kinetic parameters and affinity determination of plant-produced and commercial nivolumab binding to human PD1 protein.

Ligand	ka (1/Ms)	kd (1/s)	KD (M)
Nivolumab ΔXF	1.68e + 05	2.48e-03	1.47e-08
Nivolumab commercial	1.31e + 05	1.63e-03	1.25e-08

**Figure 7:**
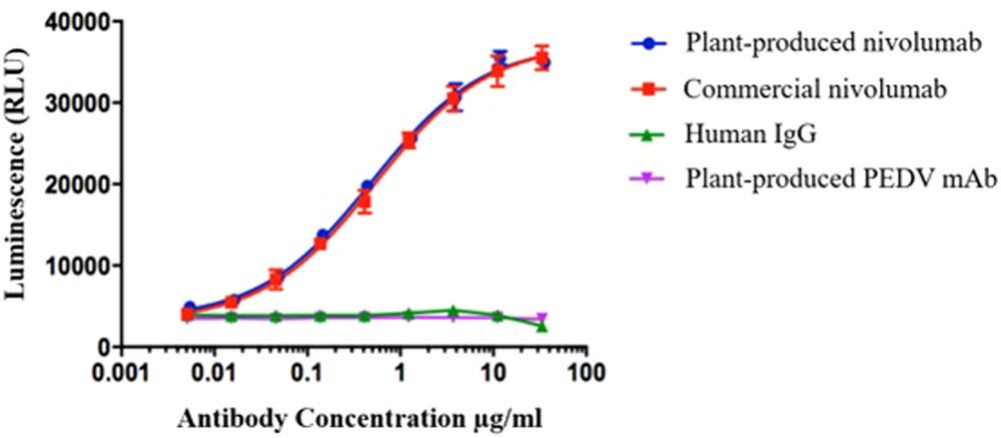
Plant-produced nivolumab blocks the PD-1/PD-1 interaction. The luminescence signal (RLU, relative light unit) at various concentrations starting from 33.33 µg/ml to 5.08 ng/ml of plant-produced nivolumab, commercial nivolumab, human IgG, and plant-produced PEDV mAb^14^ from three independent experiments are presented as the mean ± SD.

Using a luciferase reporter system, where luciferase expression is under the control of an nuclear factor of activated T-cell (NFAT) promoter, plant-produced anti-PD1 mAb induced concentration-dependent NFAT activation, similar to the commercial mammalian cell-produced antibody, with EC_50_ values of 496 ng/ml and 544 ng/ml, respectively, being observed (Fig. 7). Human IgG was used as a negative control in this study.

**Figure 8:**
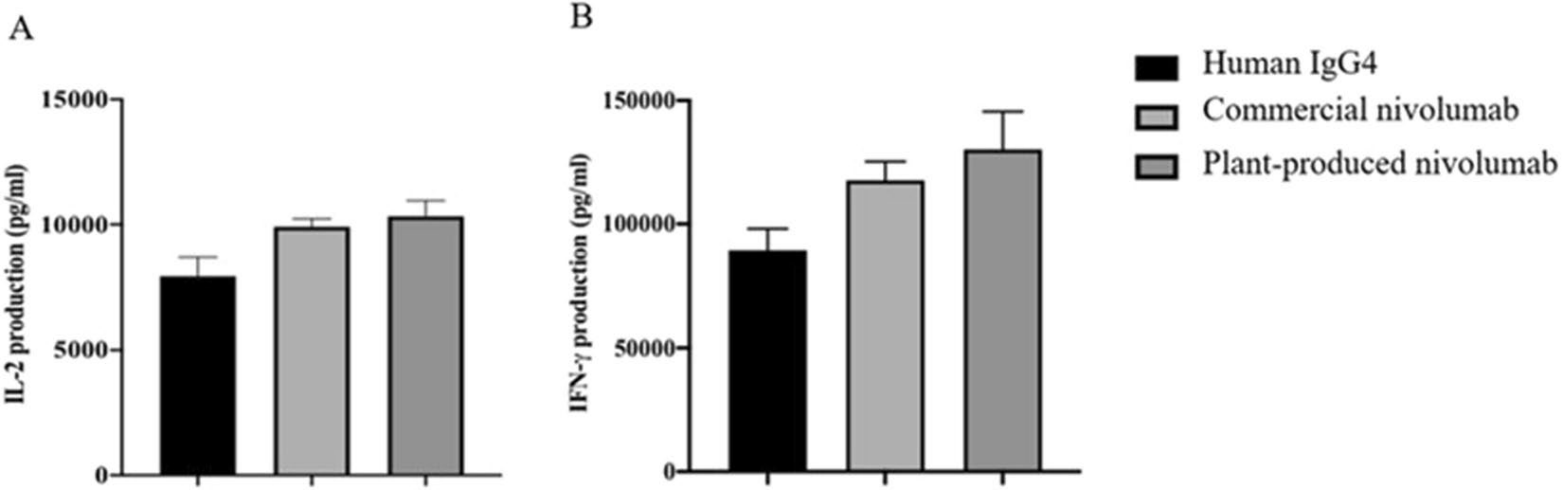
PD-1 blockade enhances T cell responses. PBMCs from 4 healthy donors were stimulated with 1 ng/ml Staphylococcal enterotoxin B (SEB) in the presence of 1 μg/ml plant-produced nivolumab, commercial nivolumab and human IgG4 antibody. The media was collected after 3 days for measurement of IL-2 (**A**) and IFN-γ (**B**) by ELISA. The amount of IL-2 and IFN-γ from three independent experiments are presented as the mean ± SD.

The ability of plant-produced anti-PD1 mAb to promote T cell responses was measured *in vitro*. The human PBMCs were stimulated by the Staphylococcal enterotoxin B in the presence of 1 μg/ml anti-PD1 mAb. Plant-produced nivolumab enhanced both IL-2 (Fig. 8A) and IFN-γ (Fig. 8B) secretion, comparing to human IgG4 negative control. In addition, the levels of IL-2 and IFN-γ enhancing from the activation of plant-produced nivolumab are not significantly different from commercial nivolumab. These experiments showed that plant-produced anti-PD1 mAb can bind potently to human PD1 protein, block the PD-1/PD-L1 interaction, and activate T cell response.

## Discussion

Immunotherapy is an attractive treatment for many cancer patients. In particular, treatment with monoclonal antibody (mAb)-based immune checkpoint blockade enhances the anti-tumour function of T cells, yielding promising results in clinical studies^8–11^. However, immunotherapy is highly expensive, which could reach up to 1 million US dollars per patient^12^. Therefore, most people in developing countries cannot access this treatment. Plant platform was developed recently for producing Our goal is to develop an alternative platform for producing effective immune checkpoint blockade mAb. In this study, we investigated the production of anti-human PD1 mAb in plants. Our results show that plant-derived anti-PD1 nivolumab mAb has similar structure and binding potency compared to mammalian cell-produced mAb. Moreover, the plant platform can address the cost and scalability issues associated with antibody production.

Transgenic plants are still prohibited in many countries^13^. To avoid concerns about genetically modified plants, transient expression technology was used in this study. Plants were recently developed to produce several monoclonal antibodies, which mostly are infectious therapeutic antibodies^15–17^ and anti-cancer antibodies^18–20^. Our study firstly showed the development of plant-produced IgG4 monoclonal antibody for immunotherapy. The nivolumab heavy chain and light chain were modified by codon optimization based on the *Nicotiana benthamiana* codon bias. The sequence Ser-Glu-Lys-Asp-Glu-Leu (SEKDEL), the endoplasmic reticulum (ER) retention motif, was added to the C-terminus of the heavy chain. The SEKDEL motif has been shown to increase the recombinant protein levels for some antibodies^21^. Presumably because the ER is a favorable compartment for protein storage as it lacks proteases that may degrade the recombinant protein.

The nivolumab was rapidly expressed in *N. benthamiana* leaves within 6 days of infiltration and successfully assembled into a native IgG monomer form (Fig. 1C,G). With genetic optimization and ER retention, mAb accumulated at an average of 140 µg/g of fresh leaf weight (Fig. 2). Previous study was shown that the PD1 binding of commercial nivolumab is highly potent^14^. Compared to the commercial mAb, both ELISA and SPR techniques showed that plant-derived nivolumab has equivalent binding kinetics *in vitro*.

The N-linked glycosylation of proteins in plants is generally similar to that in mammalian cells, but plants contain specific xylose and fucose residues that are not found in mammals^22^. Therefore, there is concern about the immunogenicity of plant-specific N-glycans in humans. The clinical data on the immunogenicity of plant N-glycans are still controversial^16–18^. However, several studies showed there is no cases with allergic or hypersensitivity symptoms^9,23–25^. For immune checkpoint blockade purposes, the IgG4 subtype was used because the induction of ADCC by IgG4 is notably weak or nonexistent^19^. This study aims to compare the anti-PD1 mAb produced in plant and mammalian cells.

Therefore, glyco-modified plants that almost completely lack β1,2-xylose and core α1,3-fucose residues on N-glycans were used in this study^20^. The N-glycan structure attached to the conserved N-glycosylation site in the heavy chain of nivolumab produced in these plants is not identical to mammalian cell-produced nivolumab (Fig. 3). In particular, the plant-produced antibody lacks core fucosylation and terminal galactosylation and displays oligomannosidic N-glycans (Man5-Man9). The oligomannosidic N-glycans are the result of SEKDEL-mediated retention in the endoplasmic reticulum (ER). The glycan variation in the Fc region of the IgG affects the binding of the Fc receptor and the antibody-dependent cell-mediated cytotoxicity (ADCC)^26,27^ and the anti-PD1 IgG4 with oligomannosidic N-glycans may also be subjected to faster *in vivo* clearance due to binding to the mannose receptors^28^. The ADCC of different anti-PD1 IgG4 glycosylation variants as well as the *in vivo* half-life will be analysed in future research. To this end, the anti-PD1 IgG4 will be produced in wild-type plants containing specific xylose and fucose residues, in ΔXF plants and without the SEKDEL peptide to reduce the amount of oligomannosidic N-glycans. Without the SEKDEL peptide the recombinant protein will be trafficking through the Golgi apparatus where the N-glycans are processed from oligomannosidic to complex N-glycans^22^. In addition to the glycan structure, the secondary and tertiary structures of the antibody produced from plant and mammalian cells are compared. The data from CD and NMR spectroscopy confirmed that the secondary and tertiary of both antibodies are similar (Fig. 4).

In addition to the glycan and protein structures, the binding efficacy of the antibody is critical. *In vitro* binding of both antibodies to human PD1 was analysed by ELISA and SRP (Figs 5 and 6). Our results confirm that plant-produced anti-PD1 mAb can bind to human PD1 protein approximately as well as mammalian cell-produced anti-PD1 mAb. To compare the potency and stability of both antibodies, a PD-1/PD1-L1 blockade cell-based bioassay (Promega, USA) was used. Interestingly, our data showed that both antibodies had similar PD-1/PD1-L1 blockade patterns at the same concentration (Fig. 7).

In summary, we established a plant cell-based platform to rapidly express and assemble anti-PD1 IgG4 mAb. Our data also show the similarities in structure and PD1 binding efficacy between plant cell-based and mammalian cell-based antibodies, supporting the potential of plant-produced anti-PD1 mAb for use in cancer immunotherapy. However, human PD1 knock-in mice are needed to study the efficacy of plant-produced anti-PD1 mAb *in vivo*. From a manufacturing perspective, the production cost of plant-produced mAb is lower than that of the mammalian system. Therefore, a plant-based alternative platform might reduce the cost of the current treatment with mAb-based immune checkpoint blockade.

## Methods

### Expression vector construction

Nucleotide sequences encoding nivolumab heavy chain (HC) and light chain (LC) were optimized *in silico* with *Nicotiana benthamiana*-optimized codons. The signal peptide was added to the N terminus, and SEKDEL was added to the C-terminus of both HC and LC genes. Optimized HC and LC sequences were synthesized (Bioneer company, South Korea). The nivolumab HC and LC plasmids were cut with *Xba*I and *Sac*I before ligation into a geminiviral vector^23^ resulting in pBYNivo-HC and pBYNivo-LC, respectively (Fig. 1A). The plasmids were transformed into *Agrobacterium tumefaciens* strain GV3101.

### Plant inoculation and protein expression

ΔXF *Nicotiana benthamiana* plants^24^ were grown in a controlled plant room with a 16 h light/8 h dark cycle at 28 °C for 6 to 8 weeks. Plants were co-agroinfiltrated with GV3101 strains containing pBYNivo-HC or pBYNivo-LC at OD_600_ 0.2 by vacuum infiltration. Infiltrated leaves were harvested on 2, 4, 6, 8, and 10 days post-infiltration (dpi) to evaluate the antibody expression pattern. For other experiments, plant leaves were harvested at 6 dpi. A blender was used to homogenize infiltrated leaves with 1x PBS (phosphate-buffered saline: 137 mM NaCl, 2.7 mM KCl, 4.3 mM Na_2_HPO_4_, 1.47 mM KH_2_PO_4_) at pH 7.4 and centrifuged at 26,000 g and 4 °C for 40 min. Crude extract was filtered with a 0.45-μm membrane filter and loaded into a protein A bead column. After washing the column with 1x PBS pH 7.4, the protein was eluted with 100 mM glycine pH 2.7 and neutralized with 1.5 M Tris base to final pH value of 7.4.

### SDS-PAGE and Western blot analysis

The commercial and purified plant-produced nivolumab antibodies were separated by sodium dodecyl sulfate polyacrylamide gel electrophoresis (SDS–PAGE) under non-reducing and reducing conditions. For non-reducing condition, the antibody was mixed with non-reducing loading solution (125 mM Tris-HCl pH 6.8, 12% (w/v) SDS, 10% (v/v) glycerol, 0.001% (w/v) bromophenol blue) and separated on 6–15% SDS–PAGE. For reducing condition, the antibody was mixed with reducing loading solution (125 mM Tris-HCl pH 6.8, 12% (w/v) SDS, 10% (v/v) glycerol, 22% (v/v) β-mercaptoethanol, 0.001% (w/v) bromophenol blue) and separated on 12% SDS–PAGE. The antibodies were either visualized by InstantBlue™ (Expedeon, UK) staining or transferred to nitrocellulose membrane (Bio-Rad, USA). The membrane was blocked with 5% skim milk and probed with HRP-conjugated anti-human gamma antiserum (The Binding Site, UK) or with HRP-conjugated anti-human kappa antiserum (The Binding Site, UK) diluted 1:5,000 in 3% skim milk. The membranes were developed by chemiluminescence using ECL plus detection reagent (Abcam, UK).

### Size exclusion chromatography

Fifty microliters of 2 µM protein solution was loaded on a Superdex 200 10/300 GL column (GE Healthcare, Little Chalfont, UK). The protein was eluted with elution buffer (50 mM Tris-HCl pH 7.5 and 150 mM KCl) at a flow rate of 0.3 ml/min. The eluted proteins were detected by the absorbance value at 280 nm.

### Antibody quantification by ELISA

The leaves harvested at 2, 4, 6, 8, and 10 dpi were extracted with 1x PBS buffer pH 7.4. A 96-well plate was coated with 50 µl of anti-human IgG-Fc fragment specific (Abcam, UK) 1:1,000 in 1x PBS buffer and incubated overnight at 4 °C. The plate was washed three times with 0.05% Tween-20 in PBS (PBST) buffer and blocked with 5% skim milk powder in 1x PBS buffer for 2 h at 37 °C. The plate was washed three times with PBST. Diluted human IgG1 kappa isotype antibody (Abcam, UK) or plant crude extracts were added into the well and incubated at 37 °C for 2 h. The plate was washed and incubated with 50 μl of HRP-conjugated anti-human kappa antiserum (The Binding Site, UK) 1:1,000 in 1x PBS buffer for 1 h at 37 °C. The plate was developed using a TMB mixture (SurModics, USA). The reaction was stopped by 50 ml of 1 M H_2_SO_4_ and measured at 450 nm with CLARIOstar (BMG Labtech, Germany).

### N-Glycan analysis

Purified nivolumab was subjected to SDS-PAGE under reducing conditions. The heavy chain was excised from the gel, S-alkylated and digested with trypsin. Liquid chromatography-electrospray ionization-mass spectrometry (LC-ESI-MS) of tryptic glycopeptides was performed as described previously^29^.

### Circular dichroism (CD) spectroscopy

Plant-produced anti-PD1 or commercial mammalian cell-produced antibodies (10 µM) were dissolved in PBS buffer (pH 7.4). CD spectra were collected using a quartz cell with a 1-mm optical path length on a J-720W CD spectropolarimeter (JASCO, Tokyo, Japan) at room temperature. The molar ellipticity expressed in degrees × cm^2^/dmol was calculated based on a mean residue molecular weight of 110. Each sample was measured in triplicate.

### NMR spectroscopy

NMR spectra were recorded on a Varian Unity INOVA 600 spectrometer (Varian, Palo Alto, CA, USA). NMR samples (100 µM) of plant-produced or commercial mammalian cell-produced anti-PD1 antibodies were dissolved in PBS buffer (pH 7.4) containing 10% v/v D_2_O. Topspin 4.0.2 software (Bruker Corp., MA) was used to process the data.

### Human PD-1 binding by ELISA

The binding ability of anti-PD-1 was evaluated by ELISA. Briefly, the MaxiSorp high protein-binding-capacity 96-well ELISA plate was coated with 10 ng/well (100 µl) of recombinant human PD-1 His tag protein at 4 °C overnight. The coated ELISA plate was washed 3 times and blocked with PBST. The twofold serial dilution of monoclonal antibodies (Plant-produced nivolumab, Commerical nivolumab^12^, Commercial atezolizumab^30^, Plant-produced PEDV mAb^14^) in PBS (starting from 200 ng/well, 100 µl) was added to the plate, incubated at 37 °C for 1 h, and then washed 3 times with PBST. The goat anti-human IgG-HRP was added (100 µL/well at a 1:10,000 dilution in PBST), incubated at 37 °C for 1 h, and washed 3 times with PBST. The OPD substrate solution (100 µL/well) (#P9187, Sigmafast™ OPD) was added to the plate and incubated in the dark at RT for 20 min. The stop solution (50 µL/well, 2 M H_2_SO_4_) was added to the plate to stop the reaction, and the absorbance at 492 nm was determined using a Cytation™ 5 cell imaging multi-mode reader.

### Kinetics/Affinity analysis by SPR technique

The surface plasmon resonance (SPR) technique was used to assess the binding kinetics/affinity of a purified plant cell-produced antibody to human PD-1 compared to a commercial antibody, nivolumab. The Biacore T200 system with a Protein G sensor chip (chip ID = 10258853) was used. For all measurements, an HBS-EP^+^ buffer consisting of 10 mM HEPES, 150 mM NaCl, 3 mM EDTA pH 7.4 and 0.005% (v/v) Tween-20 was used as the running buffer. The purified plant-produced antibody or commercial antibody was immobilized on Protein G sensor chip at 440 RU (10 µl/min, contract time 120 s). The chip surface was exposed to gradient concentrations of human PD-1 (10 nM, 20 nM, 40 nM, 80 nM, and 160 nM) at a flow rate of 30 µl/min for 60 sec. of association time and 120 sec. of dissociation time.The binding kinetics were analysed with the Biacore T200 Evaluation Software version 3.1 using a 1:1 Langmuir binding model.

### PD-1/PD-L1 neutralizing activity by PD-1/PD-L1 blockade bioassay

The neutralizing activity of the anti-PD-1 antibody was analysed by a luciferase reporter assay (#J1250, PD-1/PD-L1 Blockade Bioassays from Promega) according to the manufacturer’s instructions. Briefly, PD-L1 aAPC/CHO-K1 cells were seeded into a white flat-bottom 96-well assay plate and incubated for 16 h at 37 °C in a 5% CO_2_ humidified incubator. Serial dilutions of purified plant-produced nivolumab or commercial nivolumab were added to the plates followed by seeding PD-1 effector cells. After co-culture for 6 h at 37 °C in a 5% CO_2_ humidified incubator, the Bio-Glo™ reagent was added to the plate and incubated at ambient temperature for 5 min. The luminescence signal was measured using a Cytation™ 5 cell imaging multi-mode reader and reported as relative light units (RLUs), and the data were analysed using GraphPad Prism.

### Cytokines production by SEB stimulated PBMCs

Frozen Peripheral blood mononuclear cells (PBMCs) were isolated form healthy donors ACD blood by density gradient separation with Isoprep (Robbins Scientific Corporation, Sunnyvale, CA). PBMCs were frozen and stored in Fetal Bovine Serum (FBS) with 10% dimethyl sulfoxide (DMSO), in liquid nitrogen until assay time. On day 0, frozen PBMCs (n = 4) were thawed and seeded at 100,000 cells/well. Cells were cultured with 1 μg/ml of purified plant-produced nivolumab, commercial nivolumab and a human isotype control antibody (hIgG4, Biolegend) in triplicate. All conditions were stimulated with and without Staphylococcal enterotoxin B (SEB) at 1 ng/ml. IL-2 and IFN-γ levels in culture supernatant were performed by ELISA analysis (Biolegend, USA) on day 3.

### Statistical analysis

All three independent experiments were performed in this study. Statistical evaluation was performed data using GraphPad Prism 8.0 (GraphPad Software, San Diego, CA). A nonparametric 𝑡-test was used to determine the significance of differences between 2 groups (plant-produced nivolumab and commercial nivolumab) with non-Gaussian distributions. Statistical significance was considered to require a *P* value lower than 0.05.
